# Integrated transcriptomic and microbiota analyses reveal growth-related intestinal responses to feeding strategies in *Nibea coibor*

**DOI:** 10.1007/s44307-025-00088-2

**Published:** 2025-12-15

**Authors:** Zhaoqiu Qu, Jiayu Zhou, Ruojing Li, Qianwen Min, Xin Yi, Zhenjun Zhuang, Biao Yuan, Xubing Ba, Na Zhao, Bo Zhang

**Affiliations:** Southern Marine Science and Engineering Guangdong Laboratory-Zhanjiang, Zhanjiang, 524025 China

**Keywords:** *Nibea coibor*, Feeding strategies, Intestinal homeostasis, Microbiota, PLS-PM

## Abstract

Feeding strategies critically influence intestinal homeostasis in farmed fish, however, their underlying regulatory mechanisms remain poorly understood. *Nibea coibor*, a fish species with local characteristics in Zhanjiang (Guangdong Province, China), was chosen as the experimental model for studying feeding strategies. This study employed integrated multi-omics analyses to systematically dissect the multidimensional regulatory networks of four different feeding strategies on intestinal morphology, transcriptome, and microbiota in *Nibea coibor*. Feeding strategies reshaped gut microbiota composition and significantly altered gene expression. Compared with daytime feeding (DF), continuous fasting (CF) induced villus atrophy and goblet cell loss, disrupted microbial homeostasis (*Vibrio*, *Actinomyces*, *Photobacterium*, and *Akkermansia* upregulation), and triggered transcriptional reprogramming (*pfkfb4*, *pla2g12b*, *rptor*, and *pecam1* downregulation; *col1a* upregulation). In contrast, intermittent fasting (IF, two-day fasting/one-day feeding) achieved optimal intestinal health with the highest goblet cell density, villus height, and microbial diversity, suggesting microbiota-mediated gut plasticity and adaptation. Nighttime feeding (NF) elicited minor downregulation of energy metabolism genes without causing significant morphological or microbial alterations, indicating limited short-term circadian effects. Finally, the PLS-PM model delineated the cascade regulatory relationships linking gut microbiota, transcriptome, and intestinal morphology. These findings highlight intermittent fasting as a promising strategy to sustain intestinal homeostasis through microbiota-host synergy, while underscoring the risks of prolonged fasting-induced metabolic and barrier dysfunction. This work provides valuable insights for refining feeding protocols in marine fish aquaculture, especially *N. coibor*.

**Supplementary Information:**

The online version contains supplementary material available at 10.1007/s44307-025-00088-2.

## Introduction

Feeding strategies are pivotal in aquaculture, directly modulating growth efficiency, metabolic homeostasis, and health outcomes in farmed fish (Dar et al. 2018; Feng et al. 2025; Sun et al. 2020). Intermittent feeding strategies, through cyclic feeding-fasting phases, reduce feed costs while maintaining health in species like longfin yellowtail (*Seriola rivoliana*) (Argüello-Guevara et al. 2018) and neotropical catfish (*Lophiosilurus alexandri*) (Silva et al. 2019). Furthermore, Nile tilapia (*Oreochromis niloticus*) and Siberian sturgeon exhibit compensatory growth due to intermittent feeding (Falahatkar et al. 2025; Macêdo et al., 2021). However, prolonged fasting is associated with risks of metabolic depression (Pan et al. 2022; Pérez-Jiménez et al. 2007), growth impairment (Einen et al. 1998; Roberts et al. 2001), and intestinal atrophy (Sun et al. 2020), as documented in previous studies. By contrast, chrononutrition strategies—such as nocturnal feeding—improve nutrient utilization efficiency in species with aligned circadian rhythms, including darkbarbel catfish (*Pelteobagrus vachellii*) (Qin et al. 2017) and dabry's sturgeon (*Acipenser dabryanus*) (Chen et al. 2022). Crucially, feeding strategies shape intestinal plasticity: prolonged fasting reduces intestinal length in crucian carp (*Carassius auratus*), reversible via intermittent feeding (Li TT et al. 2019), while extended fasting intervals degrade villi structure in Nile tilapia (*O. niloticus*) (Dawood et al. 2023). Shortening of the small intestine and structural degeneration of intestinal villi are key indicators of impaired intestinal digestive function (Liu et al. 2020; Wagner et al., 2009), and this outcome directly affects the living space of goblet cells—a key type of digestive cell in the intestine (Köprücü, 2016). Despite these observations, the molecular mechanisms linking feeding schedules to intestinal adaptation remain elusive.

The gut microbiota, a metabolic interface bridging diet and host physiology (Parris et al. 2019), is highly sensitive to feeding strategies. Fasting depletes Bacteroidetes and Firmicutes, microbial phyla critical for maintaining digestive efficiency and immune homeostasis, ultimately impairing intestinal barrier integrity (Peter et al. 2020; Sakyi et al. 2020). Conversely, intermittent fasting enriches beneficial taxa (*Bacteroides*, *Akkermansia*) in crucian carp (*C. auratus*), enhancing antioxidant capacity (Li T et al. 2019). Diurnal rhythms further complicate microbial dynamics, showing species-specific succession patterns (Qin et al. 2024; Xu et al. 2022). Emerging evidence implicates microbiota-host synergy in metabolic regulation. In largemouth bass (*M. salmoides*), fasting reduces *Clostridium *sensu stricto (SCFA producer) but enriches host lipid metabolism pathways (Wang et al. 2024). Similarly, feed-restricted sea urchins (*Strongylocentrotus intermedius*) exhibit pathogenic Arcobacteraceae blooms and steroid pathway activation, increasing disease susceptibility (Ye et al. 2024). The gut-liver axis further mediates systemic responses: *Bidens pilosa L* supplementation in Nile tilapia(*O. niloticus*) reshapes microbiota to upregulate amino acid/lipid metabolism and boosts growth (Chen et al. 2024). The integration of multi-omic analyses can provide profound insights into the diversity and distribution of microbial genes (Palazzotto and Weber 2018), as evidenced by its application in studies on black tiger shrimp (*Penaeus monodon*): This approach links the abundance of the phylum Firmicutes to the host’s nutrient metabolism genes and metabolites, suggesting microbial-driven energy allocation (Uengwetwanit et al. 2020). Transcriptomic insights reveal starvation-induced metabolic reprogramming: largemouth bass (*M. salmoides*) downregulates intestinal immunity genes while activating fatty acid degradation during fasting (Wang et al. 2024). Short-term fasting-refeeding disrupts lipid and steroid metabolism (Liu et al. 2022; Qian et al. 2016), while nocturnal feeding alters retinol pathways in Chinese mitten crab (*Eriocheir sinensis*). Notably, intermittent fasting in fish lacks mechanistic characterization, contrasting mammalian studies showing mTOR suppression and enhanced autophagy (Xu et al. 2022). These findings highlight the microbiota’s role in nutrient partitioning, yet its crosstalk with host transcriptional networks under varied feeding strategies is poorly understood.

The *Nibea coibor*, a commercially vital species in Indo-Pacific waters (Huang et al. 2017a), is lauded for its rapid growth and aquaculture potential (Huang et al. 2017b; Zou et al. 2019). Existing research focuses on feed optimization (Li Z et al. 2019; Lin et al. 2021) and swim bladder collagen (Lin et al. 2023; Rong et al. 2022), neglecting feeding strategy impacts on its metabolic-immune axis. Here, we employed transcriptome and microbiota sequencing to dissect how four feeding strategies modulate growth, metabolism, and intestinal homeostasis in *N. coibor*. By integrating multi-omics analyses, this study unveiled mechanistic links between feeding schedules, microbial ecology, and host adaptive phenotype, offering actionable strategies for sustainable cultivation of this economically pivotal species.

## Materials and methods

We have read the policies on animal experimentation (ARRIVE and PREPARE guidelines) and confirm that this study complies with them. All experiments in this study were conducted with the approval of the Southern Marine Science and Engineering Guangdong Laboratory-Zhanjiang Committee.

### Daily management and sampling of fish

The juvenile *N. coibor* utilized in this investigation were sourced from marine aquaculture rafts located on Donghai Island, Zhanjiang City, Guangdong Province, China. For the fish used in the experiment, the average body weight was recorded as 13.92 ± 0.07 g, while their average body length was roughly 8–13 cm. They were temporarily reared in cylindrical containment units (500 L capacity, maintained at 30 °C water temperature with 27‰ seawater salinity). A formulated diet (Sea Horse brand, Fuzhou Seahorse Feed Co., Ltd.) was provided three times daily at 2% of the total biomass. Experimental design comprised one control group (diurnal feeding regime, DF) and three experimental cohorts: intermittent fasting with two-day feeding intervals (IF), continuous fasting (CF), and nighttime feeding (NF). 15 two-month-old fish were randomly assigned per group, with three replicates (5 fish/replicate) housed separately in 100-L glass aquarium. The filtration device is maintained every two days to ensure clean water quality. After the 21-day experimental period, to facilitate the statistical analysis of phenotypic traits, the researchers randomly selected three fish from group A, three from group B, and four from group C (three replicates in total), with each case group yielding a total of ten random samples. These fishes were euthanized via eugenol immersion (S30253-100 ml, Shanghai Yuanye Bio-Technology Co., Ltd). To ensure consistency across replicates, three fish were ultimately randomly selected from Tank C for subsequent analysis. Intestinal tissue samples were collected and processed as follows: One segment was preserved in Bouin's Fixative Solution (PH0976, Phygene), while the remaining tissues were immediately flash-frozen in liquid nitrogen and subsequently stored at −80℃.

### Histological analysis of intestine tissue

Fixed intestinal tissues specimens underwent sequential gradient alcohol dehydration using a tissue processor (JJ-12 J, Wuhan Junjie Electronics Co.), followed by embedding in molten paraffin. Trimmed paraffin blocks were sectioned at a thickness of 4 μm with a rotary microtome (RM2016, Leica Biosystems). Sections were subsequently stained using hematoxylin and eosin (H&E) protocols. Histological imaging was performed under a light microscope (Leica Application Suite X) at 200 × magnification. Quantitative analysis of villus height (VH), muscularis thickness (MT), and goblet cell density (GC) was conducted using ImageJ software (Version 1.53). Graphical representations were produced using GraphPad Prism 8.0.1.

### 16 s rRNA sequencing and analysis

Total genomic DNA was extracted from 300 mg *N. coibor* whole intestinal tissues using the TGuide S96 Magnetic Soil/Stool DNA Kit (Tiangen Biotech (Beijing) Co., Ltd.). The hypervariable V3-V4 regions of bacterial 16S rRNA genes were amplified using primer pair 338 F (5'-ACTCCTACGGGAGGCAGCA-3') and 806R (5'-GGACTACHVGGGTWTCTAAT-3'). PCR products were verified by agarose gel electrophoresis and subsequently purified using the Omega DNA Purification Kit (Omega Bio-Tek, Norcross, GA, USA). Purified amplicons were pooled and subjected to paired-end sequencing (2 × 250 bp) on the Illumina NovaSeq 6000 platform (Beijing Biomarker Technologies Co., Ltd., China). Sequence clustering was performed using USEARCH v10.0 with a 97% similarity cutoff for operational taxonomic unit (OTU) designation. Taxonomic classification of OTUs was achieved through the QIIME2 naive Bayes classifier (version 2020.6.0) (Bolyen et al. 2019), referencing the SILVA SSU rRNA database (Release 138) (Quast et al. 2012) with a minimum confidence threshold of 70%. The *α*-diversity indices (Shannon index and Simpson index) were calculated using QIIME2 to assess within-sample microbial diversity. *β*-diversity was evaluated via principal coordinate analysis (PCoA) based on the Bray–Curtis dissimilarity matrix. Statistical comparisons between groups were performed using one-way analysis of variance (ANOVA) followed by Tukey's post hoc test (*α* = 0.05). Differentially abundant microbial taxa across groups were identified using linear discriminant analysis effect size (LEfSe), with a logarithmic LDA score threshold set at > 3.0 for biomarker significance. All analyses were performed through the BMKCloud bioinformatics platform (Biomarker Technologies Corporation, Beijing; https://www.biocloud.net).

### Transcriptome sequencing

To eliminate individual differences and sample heterogeneity, three samples per group were pooled for sequencing. The number of sequenced samples was adequate for the scope of this study, with the sequencing results to be validated by subsequent qRT-PCR analysis. Total RNA was isolated from 300 mg *N. coibor* whole intestinal tissues using the TransZol™ UP Plus RNA Kit (ER501-01-V2, Beijing TransGen Biotech Co., Ltd.), followed by reverse transcription with the PrimeScript® RT Reagent Kit and gDNA Eraser (DRR047A, Takara Biomedical Technology, Beijing). RNA concentration and integrity were quantified using a Nanodrop 2000 spectrophotometer (Thermo Fisher Scientific) and the Agilent Bioanalyzer 2100 system (Agilent Technologies, CA, USA), respectively. mRNA purification was conducted using AMPure XP magnetic beads (Beckman Coulter, Beverly, USA). cDNA library preparation was performed with the Hieff NGS Ultima Dual-mode mRNA Library Prep Kit for Illumina (Panaro et al. 2000), with preliminary quantification executed on a Qubit 3.0 Fluorometer. Library fragment size distribution was verified using the Qsep400 High-Throughput Analysis System (Lu et al. 2023). Sequencing was conducted on an Illumina NovaSeq 6000 platform, with raw data processed through BMK Cloud’s bioinformatics analysis pipeline (www.biocloud.net) (Sun et al. 2017). High-quality reads (clean data) were aligned to the *N. coibor* reference genome to generate mapped datasets. Transcript abundance was quantified using FPKM normalization (Fragments Per Kilobase of transcript per Million mapped reads) (Trapnell et al. 2010). Differentially expressed genes (DEGs) were identified via DESeq2 (Love et al. 2014) with stringent thresholds (|log2(fold change)|> 1 and *p-value* < 0.05). Functional annotation of DEGs was performed against multiple databases: the NCBI Non-Redundant Protein Database (NR), Clusters of Orthologous Groups (COG/KOG), Gene Ontology (GO; http://www.geneontology.org/) (Alterovitz et al. 2007), KEGG Orthology (KO; http://www.genome.jp/kegg/) (Kanehisa et al. 2002), Pfam, and Swiss-Prot. GO term enrichment analysis was implemented using the Wallenius non-central hypergeometric test within the R package clusterProfiler v.4.4.4 (Young et al. 2010). KEGG pathway enrichment was assessed through the KOBAS database and clusterProfiler v.4.4.4 (Mao et al. 2005). Through trend analysis (conducted on the OmicShare online platform: www.omicshare.com/tools), the dynamic expression trends of genes were simulated. Genes with similar expression trends were identified and clustered into gene modules. The input data underwent standardized pre-processing via log2 transformation. With 20 predefined trend categories configured, statistically significant expression trend modules (*p* < 0.05) were annotated.

### Integration analysis

Pathway enrichment analysis revealed significantly enriched KEGG pathways (FDR < 0.05) through hypergeometric testing, identifying associated differentially expressed genes (DEGs). Second, bivariate correlation analysis (Pearson) was conducted between metabolic traits, gut microbiota parameters, and host variables, including both DEGs and top 80 microbial taxa ranked by abundance was performed using SPSS Statistics 24.0, with significant correlations (*p* < 0.05) visualized via clustered heatmaps on the OmicShare platform (https://www.omicshare.com/). Third, network analyses included KEGG pathway-DEG interaction networks constructed through OmicShare's integrated Cytoscape module and microbial co-occurrence networks generated via CNSknowall (https://cnsknowall.com/) platform with SparCC correlation thresholds (|r|> 0.8, *p* < 0.01). Finally, using the OmicShare online platform, the relative expression patterns of significantly correlated DEG-microbial pairs were visualized through Z-score normalized heatmaps through OmicShare analytical modules. Concurrently, genes and microbes were annotated as explanatory and response variables, respectively, and redundancy analysis (RDA) was performed. To better elucidate the relationships among gut microorganisms, transcriptional expression, and phenotypic traits serum glucose (GLU), cortisol (COR), lactate (LAC), and weight gain rate (WGR), we incorporated findings from a prior study (Qu et al. 2025) and conducted the same correlation analysis between gut microorganisms and differentially expressed genes (DEGs) as applied in this research.

### Validation of DEGs by qRT-PCR

To validate the reliability of the sequencing results, qRT-PCR was employed. Total RNA (from the sequencing RNA sample) was isolated using the TransZol Up Plus RNA Kit (TransGen Biotech, Beijing; ER501-01-V2) according to the manufacturer's specifications, followed by cDNA synthesis through reverse transcription using the PrimeScript™ RT reagent kit (Takara Bio, Beijing; DRR047A). Key differentially expressed genes (DEGs) identified through integrated analysis included *col1a*, *pla2g12b*, *lcat*, *itgal*, *pecam1*, *far*, *pfkfb4*, *eef1al*, *rabb3b*, and *rptor*. Gene-specific primers for qRT-PCR validation were designed using Primer Premier 5.0 (Premier Biosoft International), with sequences detailed in Table S1. Quantitative PCR amplification was performed on a CFX Opus 6 Real-Time PCR System (Bio-Rad Laboratories) with the following reaction parameters: amplification reactions were performed in 10 μL volumes containing, 5 μL ChamQ Blue Universal SYBR qPCR Master Mix (Vazyme Biotech Co., Ltd, Q312-02), 1 μL cDNA template, 0.4 μL each of forward and reverse primers (10 μM), 3.2 μL RNase-free water. Thermal cycling conditions included pre-denaturation at 95℃ for 30 s, 40 cycles of denaturation at 95℃ for 5 s, annealing at 60℃ for 10 s, and incubation at 72℃ for 10 s. Relative gene expression levels were calculated using the 2^−ΔΔCt^ method, with β-actin serving as the endogenous reference gene for normalization. All reactions were performed in technical triplicates.

### Partial least squares path modeling (PLS-PM)

In this study, intestinal morphological features, transcriptomic gene expression profiles, and gut microbial community changes were integrated with previously published data (Qu et al. 2025) on serum biochemical indices and growth performance of *N. coibor*. To explore the complex interactions among these variables, Partial Least Squares Path Modeling (PLS-PM) was applied. The model was constructed based on the following theoretical assumption: feeding strategies modulate the gut microbiota of *N. coibor*, which in turn influences host gene expression, thereby reshaping intestinal phenotype and altering serum biochemical parameters, ultimately affecting growth performance. Model fit was assessed using the Goodness-of-Fit (GoF) index, and path coefficients and coefficients of determination (*R*^*2*^) were calculated using the PLS-PM package (1000 permutations). In Partial Least Squares-Discriminant Analysis (PLS-DA), the PERMANOVA method based on the Bray–Curtis distance matrix was employed to test the compositional differences among groups.

### Data analysis

The normality of experimental data was assessed using the Shapiro–Wilk test, while the homogeneity of variances was verified through Levene's test. Significant differences (*p* < 0.05) in intestinal phenotypic indices were evaluated using Student's t-test. Gene expression levels were analyzed by one-way ANOVA (*p* < 0.05), followed by post-hoc Tukey's Honestly Significant Difference (HSD) test. All statistical procedures were implemented in SPSS 24.0.

## Results

### Intestinal morphological changes under different feeding strategies

**Fig. 1:**
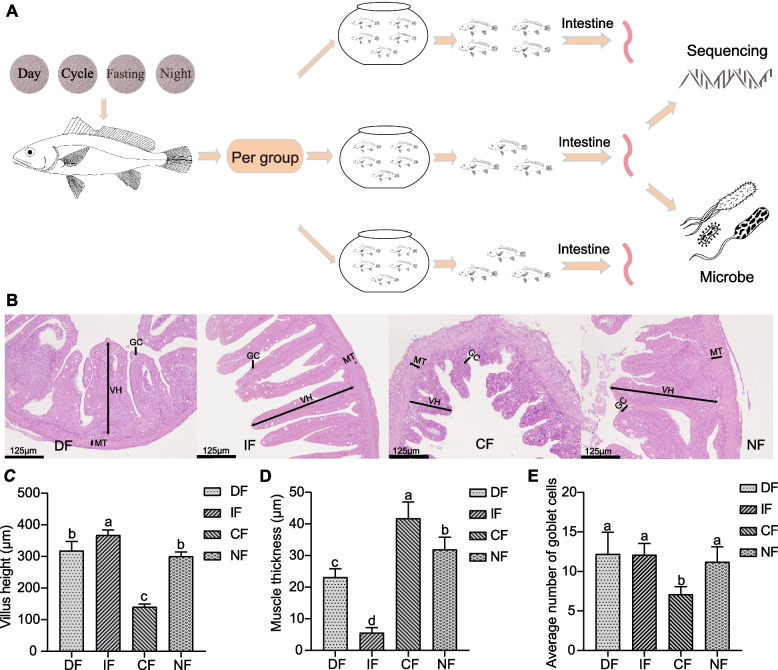
Intestinal histomorphology and phenotypic indicators across four feeding strategies in *N. coibor*. **A** A general scheme of the experimental design. **B** Representative hematoxylin–eosin (HE) stained intestinal Sects. (200 × magnification) from each experimental group. Key morphological features are labeled: VH (villus height), MT (muscle thickness), and GC (goblet cells). In panels C-E, the abscissa denotes treatment groups, while the ordinate indicates measured parameters. Distinct lowercase letters denote significant differences among groups (*p* < 0.05). **C** Comparative analysis of villus height among groups. **D** Muscle thickness measurements across treatment groups. **E** Quantification of goblet cell density per intestinal villus

The intestinal architecture of *N. coibor* exhibited distinct morphological changes in response to varying feeding strategies (Fig. 1A). Quantitative analysis revealed significant variations in villus height (VH), with the CF group exhibiting markedly reduced VH compared to other groups (*p* < 0.05), while IF induced a significant increase in VH (*p* < 0.05). No statistically discernible differences were observed between the DF and NF groups (Fig. 1B). Muscularis thickness (MT) demonstrated significant intergroup heterogeneity (Fig. 1C), with CF displaying the highest MT values and IF showing the lowest (*p* < 0.05). Notably, NF exhibited significantly greater MT than DF (*p* < 0.05). Goblet cell density (GC) was substantially diminished in the CF group relative to all other cohorts (*p* < 0.05), while no significant differences in GC were detected among the DF, IF, and NF groups (Fig. 1D).

### Feeding strategy-induced alterations in microbial community composition

**Fig. 2:**
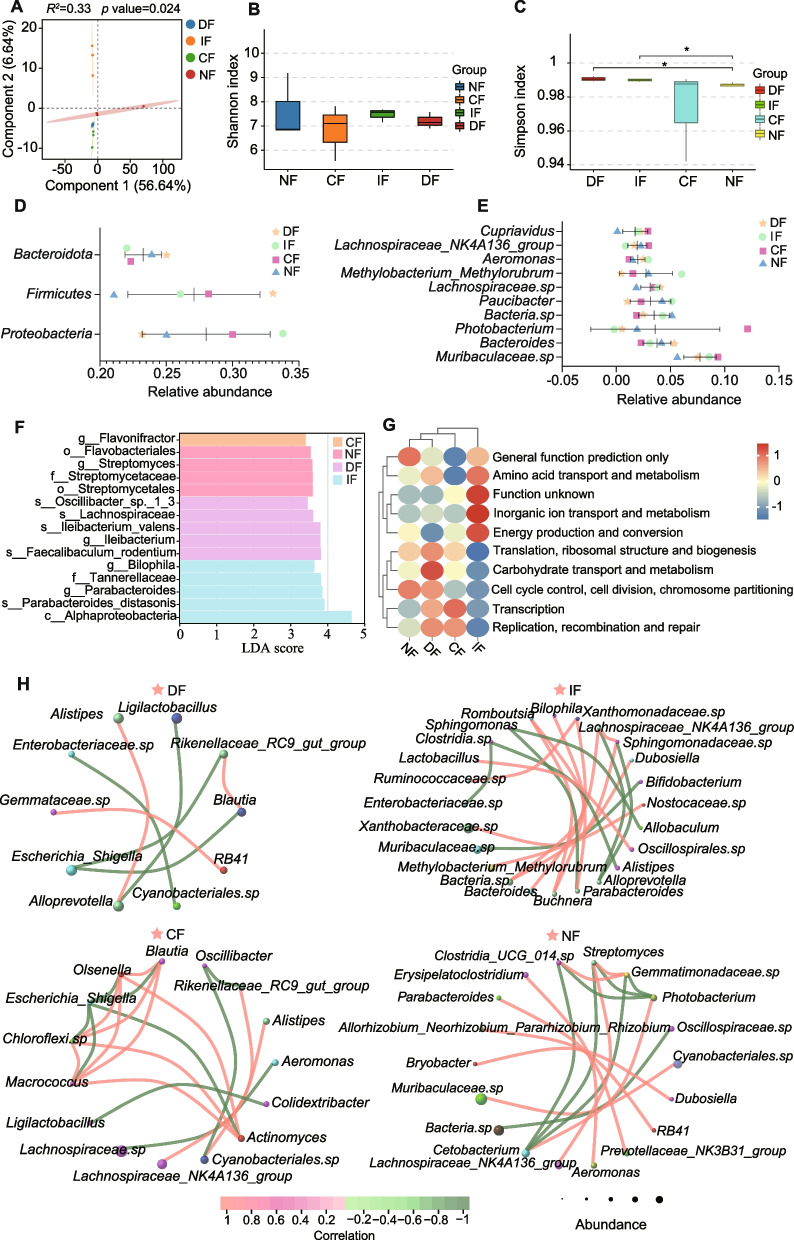
Gut microbiota profiling and diversity analysis in *N. coibor* under different feeding strategies. **A** Partial Least Squares Discriminant Analysis (PLS-DA), where the abscissa and ordinate represent the first and second component scores, with colors distinguishing experimental groups. Alpha diversity indices: Shannon index (**B**) and Simpson index (**C**), comparing microbial diversity (abscissa: groups; ordinate: index values). Taxonomic composition at the phylum (**D**) and genus (**E**) levels: the abscissa indicates relative abundance, and the ordinate lists taxonomic classifications, with colors/symbols denoting group-specific distributions. **F** LEfSe results, the abscissa shows LDA scores (log10), and the ordinate displays taxonomic ranks of discriminant taxa. **G** Functional prediction via PICRUSt2 with COG annotation (abscissa: groups; ordinate: functional categories). **H** Microbial co-occurrence network: node size corresponds to taxon abundance, lines represent Pearson correlations (red: positive; green: negative), with line thickness reflecting correlation strength

The genus-level PLS-DA model (Fig. 2A) revealed clear separation among four groups, and similar distinct separations were also observed at the phylum and OTU levels (Fig. S1). Alpha diversity metrics showed significant microbial community alterations induced by irregular feeding strategies, as evidenced by Shannon and Simpson indices (Fig. 2B-C). Notably, the Simpson index indicated statistically significant differences (*p* < 0.05) in community composition between the NF group and both DF/IF groups. At the phylum level (Fig. 2D), Proteobacteria, Firmicutes, and Bacteroidota were identified as the dominant microbial taxa. Specifically, the relative abundance of Firmicutes and Bacteroidota in the DF group was higher than that in other groups, while the relative abundance of Proteobacteria was lower. At the genus-level, *Muribaculaceae sp*., *Bacteroides*, and *Photobacterium* were the dominant intestinal microbial taxa among groups; in particular, the relative abundance of Photobacterium and Muribaculaceae sp. in the CF group was higher than that in other groups, whereas the relative abundance of Bacteroides was lower. (Fig. 2E). Feeding strategies significantly modulated their relative abundances. LEfSe analysis (Fig. 2F) revealed distinctive microbial signatures: intermittent fasting markedly enriched Alphaproteobacteria, Tannerellaceae, Parabacteroides, and Bilophila (LDA score > 3.0), while continuous fasting preferentially enhanced Flavonifractor populations. COG functional prediction analysis (Fig. 2G) demonstrated the functional shifts induced by feeding strategies, primarily involving ribosomal structure and biogenesis, carbohydrate transport and metabolism, replication, recombination and repair, transcription, cell cycle control, cell division, chromosome partitioning, inorganic ion transport and metabolism, amino acid transport and metabolism, and energy production and conversion. Microbial co-occurrence network analysis (Fig. 2H) revealed altered community complexity across groups, with the DF group exhibiting simplified network topology compared to other groups, suggesting enhanced microbial interactions under modified feeding protocols.

### Fasting inhibits signaling pathways associated with fatty acid metabolism

**Fig. 3:**
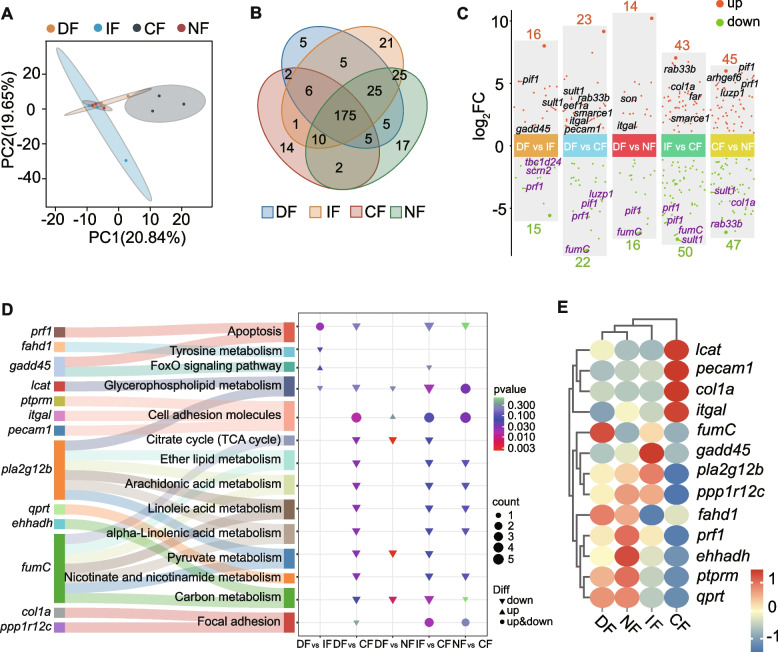
Comparative transcriptomic analysis across four feeding strategies in *N. coibor*. **A** Principal component analysis (PCA) plot. Orange corresponds to daytime feeding (DF), blue to intermittent fasting (IF), gray-green to continuous fasting (CF), and red to nighttime feeding (NF). The abscissa and ordinate represent scores of the first and second principal components, respectively. **B** Venn diagram of shared and unique genes. Color assignments: red (CF), blue (DF), orange (IF), and green (NF). **C** Volcano plots of differentially expressed genes (DEGs). the abscissa indicates different comparison groups, and the ordinate shows log2(fold change). Upregulated and downregulated genes are highlighted in orange-red and green, respectively. **D** KEGG pathway enrichment bubble plot. The abscissa denotes comparison groups, while the ordinate lists enriched KEGG pathways with associated DEGs. Color intensity reflects statistical significance, symbol size indicates enrichment degree (gene ratio), and arrow direction denotes regulation trends (upward: upregulation; downward: downregulation). **E** Heatmap of DEG expression profiles. rows represent experimental groups. Columns correspond to individual genes. Upregulated and downregulated genes are colored red and blue, respectively

Principal component analysis (PCA) revealed distinct separation of the CF group from other experimental groups, while incomplete differentiation was observed among the DF, CF, and NF groups (Fig. 3A). Differential expression analysis identified five, 21, 14, and 17 unique differentially expressed genes (DEGs) in the DF, IF, CF, and NF groups, respectively (Fig. 3B). Multi-comparison volcano plot analysis demonstrated significant transcriptional alterations: compared to DF, IF exhibited 16 upregulated and 15 downregulated genes; CF showed 23 upregulated and 22 downregulated genes, while NF displayed 14 upregulated and 16 downregulated genes (Fig. 3C). Subsequent pairwise comparisons revealed 43 upregulated and 50 downregulated genes in CF versus IF, along with 45 upregulated and 47 downregulated genes in NF relative to CF. Series Test of Cluster analysis of DEGs revealed three significantly enriched expression profiles: Profile 11 (containing 53 genes), Profile 8 (containing 53 genes), and Profile 4 (containing 32 genes). The four breakpoints correspond to the four groups (DF, IF, CF, and NF) respectively. Each profile demonstrates the expression differences of significantly enriched gene clusters among the four groups. Notably, the gene clusters in the CF group of Profile 11 all exhibit higher expression levels than those in the other groups, whereas the gene clusters in the CF group of Profile 8 exhibit lower expression levels than those in the other groups (Fig. S2). KEGG pathway enrichment analysis (Fig. 3D) highlighted predominant involvement in energy metabolism pathways, particularly lipid-related processes including the tyrosine metabolism, glycerophospholipid metabolism, TCA cycle, ether lipid metabolism, and fatty acid metabolism pathways (arachidonic acid, linoleic acid, and α-linolenic acid metabolism). Additional metabolic pathways encompassing pyruvate metabolism, nicotinate/nicotinamide metabolism, and carbon metabolism were significantly enriched. Furthermore, immune-related pathways, including apoptosis, cell adhesion molecules, FoxO signaling, and focal adhesion mechanisms were identified as differentially regulated. Hierarchical clustering analysis (Fig. 3E) indicated conserved expression profiles between the DF and NF groups, divergent patterns in IF compared to DF/NF, and distinct expression signatures in CF versus other groups. This indicates that the feeding strategy of the CF group may have affected energy metabolism and the immune barrier.

### Significant associations between key genes, microbes, and intestinal phenotypes

**Fig. 4:**
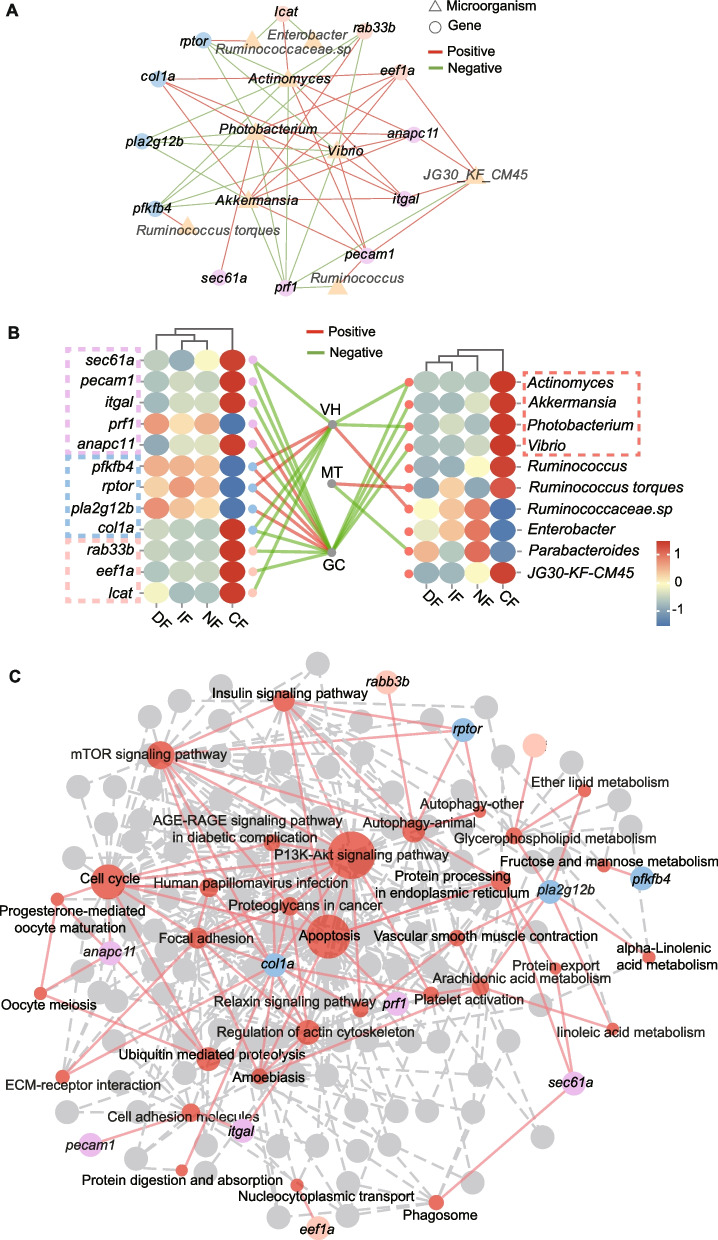
Integrative analysis of intestinal phenotypes in relation to transcriptomic profiles and gut microbiota. **A** Correlation network between genes and gut microbial taxa. Circular nodes represent genes, and triangular nodes represent microbial taxa. Edges indicate significant correlations, with red representing positive and green representing negative correlations. **B** Heatmap visualization of associations between intestinal phenotypic traits, gene expression, and microbial composition. The left heatmap displays the relative expression levels of selected genes, and the right heatmap shows the relative abundance of selected microbial taxa. Lines connecting the three intestinal phenotypes to genes or microbes represent significant correlations, with red indicating positive and green indicating negative relationships. "VH", "MT", and "GC" refer to "quantitative analysis of villus height", "muscularis thickness", and "goblet cell density". **C** KEGG pathway network of selected genes from the transcriptomic analysis. Red nodes represent KEGG signaling pathways, while orange, blue, and purple nodes stand for genes of different functional categories

Correlation analysis between intestinal morphological traits (VH, MT, GC) and differentially expressed genes (DEGs) identified 14 key genes (Fig. S3A), which were functionally classified based on KEGG pathways into immune-related genes (*pecam1*, *sec61*, *anapc11*, *prf1*, *itgal*), metabolism-related genes (*rab33b*, *eef1a*, *lcat*), and genes involved in both immune and metabolic processes (*rptor*, *pla2g12b*, *pfkfb4*, *col1a*) (Fig. S3B). Association analysis between these genes and the top 80 microbial genera identified 10 key taxa, with *Akkermansia*, *Vibrio*, *Actinomyces*, and *Photobacterium* served as the core microbial taxa in the network (Fig. 4B). Interaction mapping of VH, MT, GC with DEGs and microbial communities showed that VH was positively correlated with *rptor* and *pfkfb4*, and negatively correlated with *lcat*, *rab33b*, *sec61a*, and *col1a*; GC was positively associated with prf1, *pla2g12b*, *rptor*, and *pfkfb4*, and negatively correlated with *eef1a*, *rab33b*, *anapc11*, *itgal*, *pecam1*, *sec61a*, and *col1a* (*p* < 0.05). Additionally, VH was positively correlated with abundance of *Vibrio* and *Ruminococcaceae.sp*, and negatively correlated with *Photobacterium* and *Actinomyces*; MT showed a positive correlation with *Ruminococcus torques* and a negative correlation with *Parabacteroides*; GC was negatively associated with *Vibrio*, *Photobacterium*, *Akkermansia*, and *Actinomyces* abundance (*p* < 0.05). Heatmap visualization further revealed that both gene expression and microbial abundance in the CF group exhibited inverse trends compared to the other feeding strategies (Fig. 4C). KEGG enrichment network analysis identified *col1a* and *pla2g12b* as key genes linking lipid metabolism and immune regulation, and highlighted a central signaling pathway network involved in these processes (Fig. 4D). Finally, RDA analysis (Fig. S4A) and a dynamic network heatmap (Fig. S4B) were used to validate the correlations between these key genes and microbial taxa, supporting the robustness and consistency of the observed associations.

### Identification of key genes and microbes related to physiological traits

**Fig. 5:**
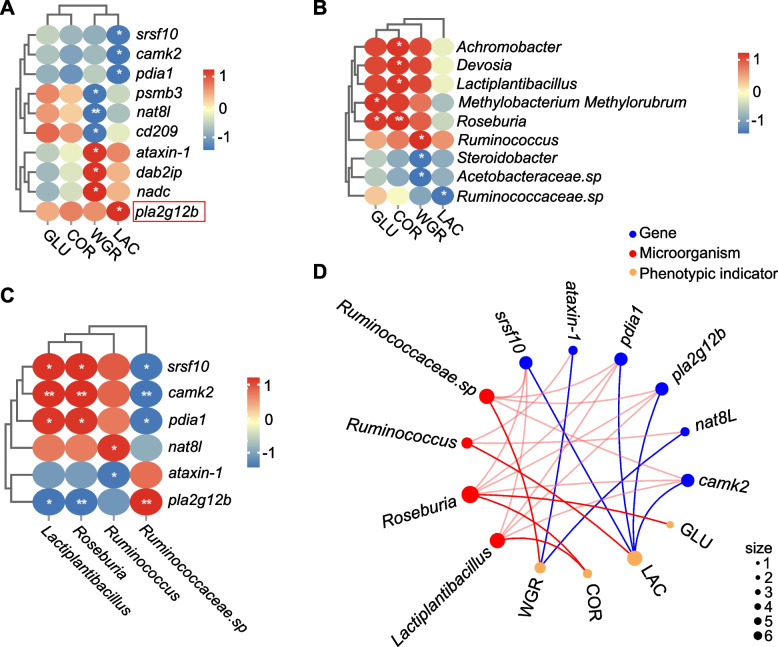
Correlation analysis of serum biochemical parameters and WGR with intestinal transcriptome and microbiota. Correlation analysis of serum biochemical parameters and weight gain rate with gene expression (**A**) and gut microbial (**B**) abundance. **C** Heatmap of significant associations between genes and microbial taxa based on correlation analysis, with non-significant relationships removed. **D** Correlation network integrating phenotypic traits, genes, and genera. Yellow nodes represent phenotypic traits; blue nodes represent genes, with blue edges indicating significant correlations between genes and traits; red nodes represent microbial taxa, with red edges indicating significant correlations with traits; light pink edges denote significant correlations between genes and microbial taxa

Correlation analysis between serum glucose (GLU), cortisol (COR), lactate (LAC), and weight gain rate (WGR) with gene expression (Fig. 5A) and bacteria abundance (Fig. 5B) identified several key genes and microbial taxa. Notably, *ataxin-1*, *dab2ip*, and *nadc* exhibited significant positive correlations with WGR, while *psmb3*, *nat8l*, and *cd209* were significantly negatively correlated with WGR (*p* < 0.05). Additionally, *pla2g12b* was positively correlated with LAC, whereas *srsf10*, *camk2*, and *pdia1* were negatively correlated with LAC (*p* < 0.05) (Fig. 5A). Similarly, *Methylobacterium Methylorubrum* and *Roseburia* were positively correlated with GLU (*p* < 0.05). *Lactiplantibacillus*, *Devosia*, *Achromobacter*, and *Roseburia* were positively correlated with COR (*p* < 0.05). *Ruminococcus* showed a positive correlation with WGR, while *Acetobacteraceae.sp* and *Steroidobacter* exhibited negative correlations with WGR (*p* < 0.05). Finally, *Ruminococcaceae.sp* was negatively correlated with LAC (*p* < 0.05). Furthermore, correlation analysis was performed between the selected genes and microbial taxa. The results demonstrated varying degrees of association between the genes (*pla2g12b*, *ataxin-1*, *nat8l*, *srsf10*, *camk2*, and *pdia1*) and the microbial species (*Ruminococcaceae.sp*, *Ruminococcus*, *Roseburia*, and *Lactiplantibacillus*) (Fig. 5C). These four metabolic traits were then integrated with the screened genes and microbes to construct a comprehensive correlation network (Fig. 5D). Most of the identified genes and microbes were significantly altered in the CF group (Fig. S5), highlighting the pronounced effects of starvation.

### Validation of screened DEGs by qRT-PCR

**Fig. 6:**
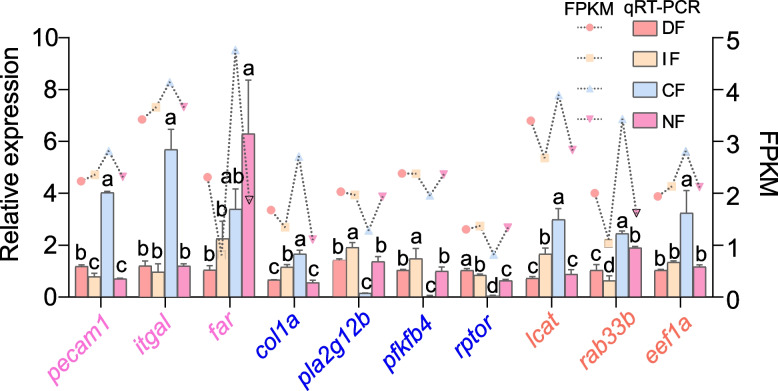
Dual-axis composite plot integrating transcriptomic and qRT-PCR validation data. The abscissa represents gene identifiers, with the left ordinate corresponding to quantitative reverse transcription PCR (qRT-PCR) results (relative expression calculated via the 2^−ΔΔCT^ method), and the right ordinate displays log10-transformed FPKM values from RNA-seq. Bar profiles (abscissa: experimental groups; ordinate: expression ratios) are color-coded as follows: pink = daytime feeding (DF), orange = intermittent fasting (IF), light blue = continuous fasting (CF), purple = nighttime feeding (NF). Superscript lowercase letters (a, b, c, d) denote statistically significant intergroup differences (*p* < 0.05). Line plots (abscissa: gene order; ordinate: normalized FPKM) overlay transcriptomic trends, with node colors matching group-specific bar plot assignments

Ten DEGs were identified through screening and subsequently validated by qRT-PCR analysis of their expression profiles (Fig. 6). The results demonstrated distinct expression patterns across groups: in the CF group, *col1a*, *lcat*, *itgal*, *pecam1*, *eef1a1*, and *rabb3b* exhibited significantly higher relative expression levels compared to other groups (*p* < 0.05), whereas *pla2g12b*, *pfkfb4*, and *rptor* showed significantly lower expression levels (*p* < 0.05). The IF group displayed elevated expression of *col1a*, *pla2g12b*, *lcat*, and *pfkfb4* relative to both the DF and NF groups (*p* < 0.05). Conversely, *pecam1* and *rabb3b* expression was significantly reduced compared to the DF group (*p* < 0.05), while *far* expression decreased relative to the NF group (*p* < 0.05). The *rptor* expression in IF showed intermediate levels, being significantly lower than DF but higher than NF. In the NF group, *pecam1* and *rptor* expression levels were significantly lower than the DF (*p* < 0.05), whereas *far* expression was markedly elevated compared to DF (*p* < 0.05). Functional analysis revealed distinct biological roles: *pla2g12b*, *lcat*, *pfkfb4*, and *far* were primarily involved in lipid metabolism regulation; *col1a*, *itgal*, *pecam1*, and *rptor* contributed to immune-metabolic crosstalk.

### PLS-PM reveals the driving effects of microbial communities, genes, and phenotypic traits on growth performance

**Fig. 7:**
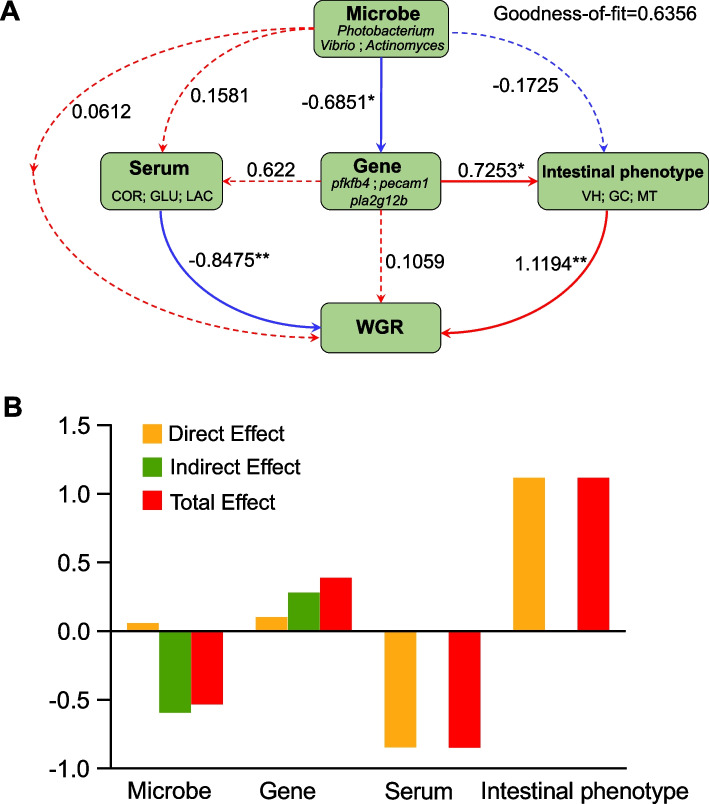
Effects of key factors on *N. coibor* growth performance revealed by PLS-PM. **A** Partial least squares path modeling (PLS-PM) was used to illustrate the cascade relationships among various factors. Each circle represents an observed or latent variable. Solid lines represent significant correlations, with the values on the lines indicating path coefficients. Statistical significance is denoted by an asterisk ‘*’ (*p* < 0.05). Red and blue lines indicate positive and negative correlations, respectively. **B** Based on the PLS-PM results, the standardized effects of each factor on the growth performance of *N. coibor* were quantified. The total effect was calculated as the sum of both direct and indirect effects

To elucidate the mechanistic pathways linking gut microbiota, host gene expression, intestinal phenotype, and serum biochemical factors to growth performance (weight gain rate, WGR), the PLS-PM framework was constructed (Fig. 7A). The model demonstrated a good overall fit (GoF = 0.6356), indicating a reliable level of explanatory adequacy. Notably, microbial composition exerted a significant negative effect on host gene expression (path coefficient = −0.685, *p* < 0.05). In turn, the expression of key genes had a significant positive influence on intestinal phenotypes (VH, GC and MT) (path coefficient = 0.7253, *p* < 0.05). These intestinal phenotypic traits were strongly associated with WGR, showing the highest direct positive effect within the model (path coefficient = 1.1194, *p* < 0.01). Serum biochemical parameters (GLU, COR, LAC) had a significant negative impact on WGR (path coefficient = –0.8475, *p* < 0.01) (Fig. 7A). Indirect pathways further highlighted that microbial suppression of genes was a key driver of reduced WGR (direct effect = 0.061, indirect effect = −0.595), whereas gene-mediated intestinal improvements positively contributed to growth (direct effect = 0.106, indirect effect = 0.285) (Fig. 7B). The model positions intestinal health as the central hub translating genetic signals into growth outcomes, with microbial influences acting upstream through transcriptional regulation.

## Discussion

Feeding strategies modulate fishes physiology by altering energy balance and gut microbiota.. Circadian rhythm-driven feeding elicits species-specific responses (Flood et al. 2011; Rubio et al. 2003). Notably, the intestine mediates these effects as both absorptive organ and microbial interface (Moore et al. 2011; Tremaroli and Backhed 2012). In *N. coibor*, a comprehensive analysis integrating intestinal morphology, transcriptome, and microbiota under four distinct feeding strategies revealed their differential impacts on intestinal homeostasis. Notably, for the transcriptome sequencing component of this multi-dimensional analysis, three samples per group are adequate to address the current research question, whereas an expanded sample size would be required for in-depth inquiry into more advanced research topics.

First, intermittent fasting (IF) demonstrated a significant advantage in maintaining intestinal structural homeostasis in *N. coibor*, a finding consistent with observations in other species. In the present study, continuous fasting (CF) led to a marked reduction in villus height (VH) and goblet cell (GC) numbers, which aligns with the established understanding that prolonged starvation induces intestinal atrophy (Krogdahl and Bakke-McKellep 2005; Su et al. 2021). Conversely, the highest villus height (VH) was observed in the IF group, suggestive of adaptive mucosal hyperplasia. This enhancement of the absorptive surface area serves to maximize nutrient assimilation upon refeeding-an evolutionarily conserved adaptive response that has been reported in Nile tilapia subjected to fasting-refeeding cycles (Dawood et al. 2023) and in mammalian models (Liu et al. 2021). Villus height (VH) and goblet cell (GC) number serve as key indicators of intestinal health (Islam et al. 2021). Their increase contributes to gut homeostasis by expanding the absorptive surface area-as demonstrated in probiotic-fed Litopenaeus vannamei (Zhang et al. 2020)—and by enhancing the chemical barrier function through mucin secretion (Kim and Khan 2013; McGuckin et al. 2011), respectively.

Furthermore, the gut microbiota, a critical factor in nutrient partitioning in fish (Chen et al. 2021), undergoes feeding strategy-specific compositional shifts that are modulated by host metabolism (Liu et al. 2020) and are critically linked to intestinal homeostasis. It is recognized that fasting-induced metabolic stress can trigger significant microbial alterations (Xia et al. 2014). Correspondingly, our study revealed that feeding regimes induced changes in microbial diversity, with these shifts being particularly evident in key phyla such as Proteobacteria, Firmicutes, Actinobacteria, and Bacteroidetes (Kormas et al. 2014). Our analysis revealed a significant enrichment of potentially pathogenic bacteria, including Vibrio, Actinomyces, and Photobacterium, in the CF group. The enrichment of Vibrio is particularly concerning, as it is well-documented to cause damage to the intestinal mucosa and epithelial cells, provoke inflammation (Fu et al. 2022), and further compromise gut barrier function (Zhou et al. 2022). Similarly, the genus Actinomyces encompasses numerous pathogenic species (Wang et al. 2021; Fu et al. 2023) and has been associated with metabolic disorders (Xie et al. 2024) and inflammatory responses (Stella et al. 2022). Furthermore, *Photobacterium* has been consistently associated with compromised health in various aquatic stress models, including diseased Litopenaeus vannamei (Yu et al. 2018), hypoxic Bostrychus sinensis (Fan et al. 2020), and Rachycentron canadum (Wang et al. 2021). Collectively, the abundances of these genera demonstrated negative correlations with villus height (VH) and goblet cell (GC) numbers, suggesting their potential involvement in fasting-induced intestinal compromise. In contrast, the IF strategy effectively maintained a healthier microbial community structure. Given that gut microbiota composition can directly regulate goblet cell proliferation via microbial metabolites (Pirarat et al. 2011; Standen et al. 2015), the IF group likely fostered a beneficial microbial environment that indirectly supported the integrity of the intestinal epithelial barrier, which is crucial for maintaining gut homeostasis in *N. coibor*.

At the molecular level, IF sustained the expression of key genes governing energy metabolism and barrier function, mechanistically explaining its capacity to maintain intestinal homeostasis. In contrast, CF resulted in the downregulation of critical energy metabolism genes (*pfkfb4, pla2g12b*), which are essential for glycolytic regulation and phospholipid metabolism, respectively (Dai et al. 2022; Drew et al. 2008; Okamura et al. 2021). Notably, our previous work (Qu et al. 2025) documented significantly reduced serum lactate levels in the CF group. Considering that *pfkfb4* serves as a master regulator of the glycolysis-lactate axis (Lu et al. 2024; Long et al. 2025; Kar et al. 2023), its observed downregulation suggests its involvement in mediating the metabolic reprogramming in response to starvation stress in *N. coibor*. Concurrently, barrier-related genes (*rptor, pecam1, col1a*) exhibited significant alterations. Under fasting conditions, the downregulation of *rptor* inhibits mTORC1 activity and impairs epithelial renewal (Wei et al. 2023). We observed that the expression of rptor exhibits a positive correlation with both villus height (VH) and goblet cell (GC) counts, and this finding aligns with the conclusion from other studies that inhibition of the mTOR pathway leads to intestinal atrophy (Xie et al. 2020). While CF elevated *pecam1* (a marker of immune adjustment; Ma et al. 2023) and *col1a* (critical for structural integrity; Frantz et al. 2010), IF maintained homeostatic expression of these genes. This suggests IF preserves epithelial turnover through mTOR pathway sustainment, molecularly explaining its morphological superiority.

In the present study, intermittent fasting (IF) in *N. coibor* resulted in enhanced microbial diversity and a more complex network architecture. Notably, IF promoted intestinal adaptation primarily through enriching microbial diversity rather than via transcriptional reprogramming. This finding aligns with observations in Eriocheir sinensis, where IF was shown to reinforce microbial stability and enhance host immune function (Li T et al. 2019). The elevated GC density and VH further reflect a microbiota-driven mucosal protection mechanism, where pathogens are suppressed through ecological competition rather than transcriptional adaptation-implying a significant role for post-transcriptional regulation. Ultimately, our PLS-PM integrative model delineates a hierarchical regulatory pathway that maintains intestinal homeostasis and promotes growth, providing a novel systemic perspective for understanding the mechanistic basis of different feeding strategies. The model revealed that while the gut microbiota does not directly determine fish growth performance (WGR), it indirectly regulates intestinal architecture by modulating host gene expression, with this architecture serving as the most immediate driver of weight gain rate. This "microbiota-gene-morphology" regulatory cascade aligns with established principles of host physiology modulation by gut microbes (Tremaroli and Bäckhed, 2012; Xia et al. 2014). These transcriptional alterations subsequently induced significant changes in intestinal structural metrics, notably villus height (VH) and goblet cell (GC) density-established markers of nutrient absorption capacity and barrier integrity (McGuckin et al. 2011). Our PLS-PM analysis confirmed that, unlike the extensive transcriptional reprogramming and microbial dysbiosis triggered by CF, IF predominantly drives healthy mucosal remodeling through microbial ecosystem stabilization. This mechanism represents a more metabolically economical strategy for maintaining systemic intestinal homeostasis. The integrated model thereby provides a solid theoretical framework for optimizing feeding strategies in Nibea coibor based on intestine-driven physiological metrics.

## Conclusion

Intermittent fasting (IF) enhanced intestinal homeostasis in *N. coibor* by promoting structural integrity and increasing microbial diversity, highlighting its potential as a sustainable aquaculture strategy for *N. coibor* aquaculture. In contrast, continuous fasting (CF) compromised gut structure and function, manifesting as microbial dysbiosis (dominated by *Vibrio*, *Actinomyces*, and *Photobacterium*), suppressed host metabolism (downregulated *pfkfb4* and *pla2g12b*), and altered intestinal morphology (downregulated *rptor* and *pecam1*, upregulated *col1a*). Nighttime feeding (NF) induced mild circadian rhythm adaptation without causing intestinal structural damage. These findings underscore the benefits of IF in maintaining gut health through host-microbiota interactions and caution against the physiological risks of prolonged fasting.

## Supplementary Information

Supplementary Material 1.

## Data Availability

Raw data have been uploaded and are available on the NCBl website (Accession NO. PRJNA1056803 and NO. PRJNA1197723).
