# Supplementary material for: Integrated transcriptomic and microbiota analyses reveal growth-related intestinal responses to feeding strategies in Nibea coibor
Source: Adv Biotechnol (Singap). 2025 Dec 15;3(4):37. doi: 10.1007/s44307-025-00088-2 (PMC12702818; doi:10.1007/s44307-025-00088-2)
Supplement: Supplementary file 1 — Supplementary Material 1. [file 44307_2025_88_MOESM1_ESM.docx]

Supplementary Table.1. Primer sequences used in this study


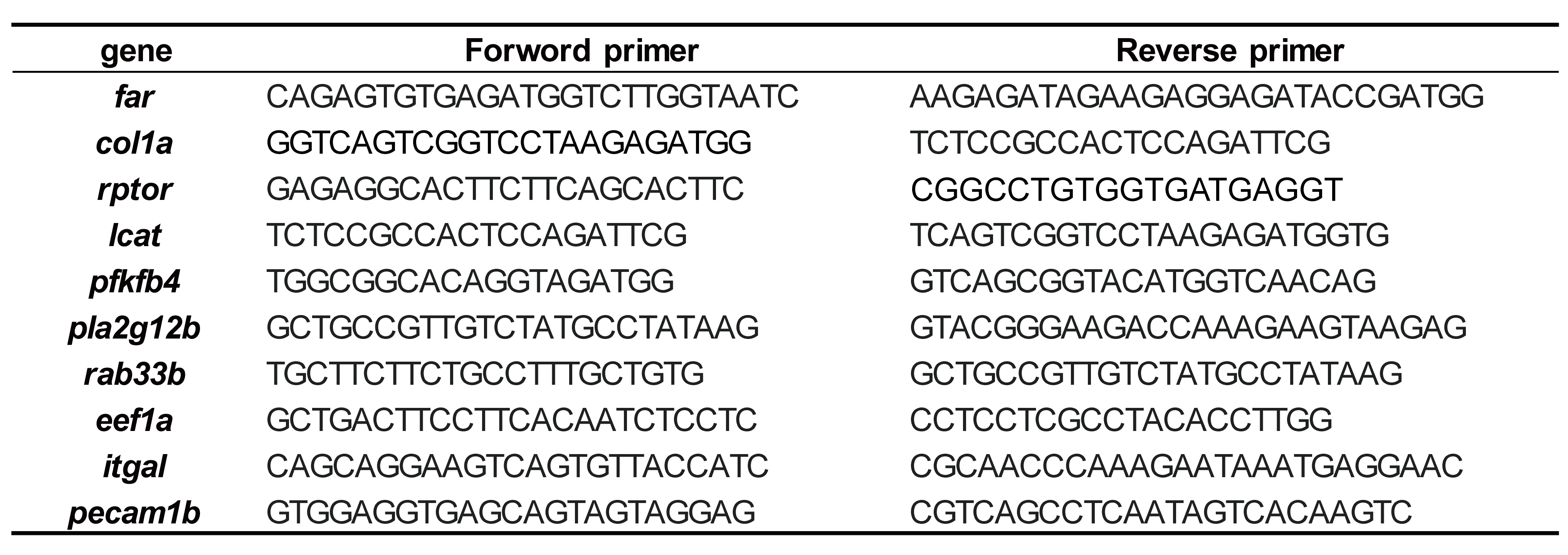


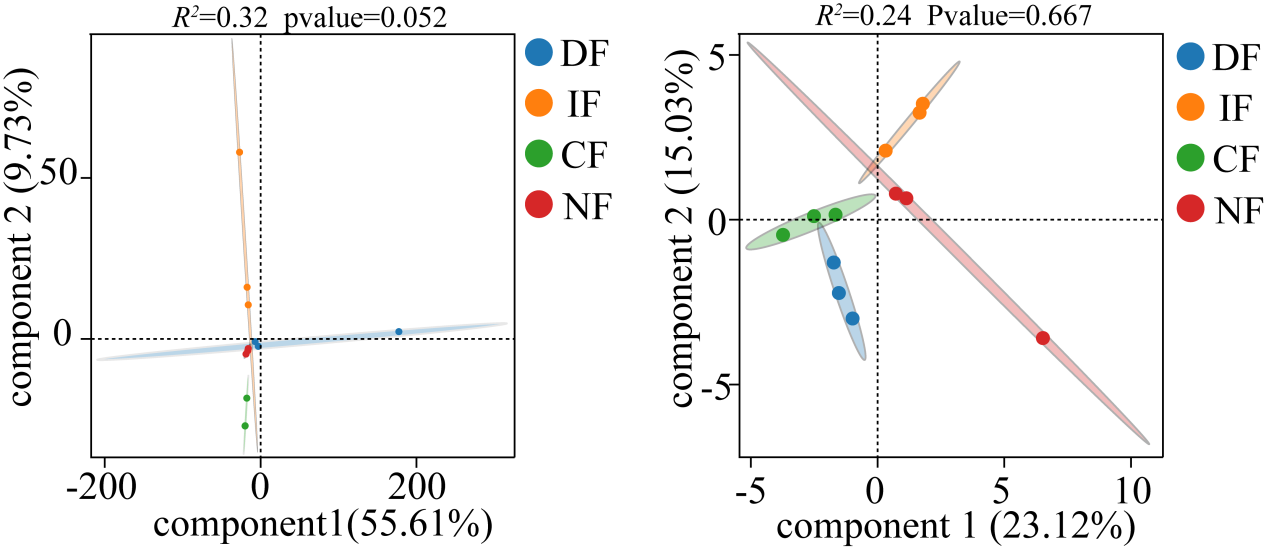


Supplementary Fig.1. Partial least squares discriminant analysis (PLS-DA) of microbial communities. The left panel shows analysis at the OTU level, while the right panel shows analysis at the phylum level. Colors indicate different experimental groups.


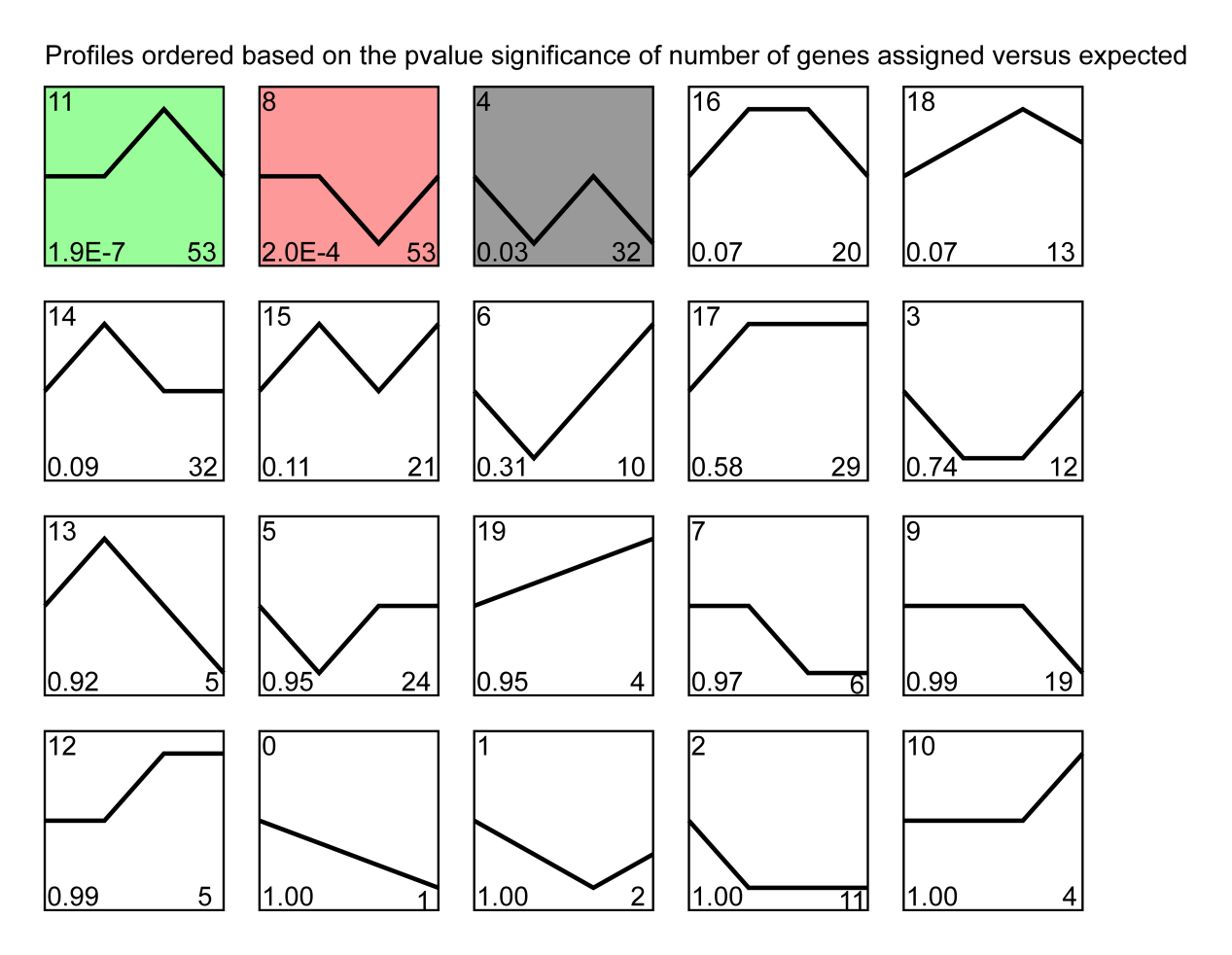


Supplementary Fig.2. Gene co-expression trend analysis from transcriptomic data. Modules represent clusters of genes with similar expression patterns. Colored modules are significantly enriched; significance values are shown in the bottom left, and the number of genes per module is shown in the bottom right.


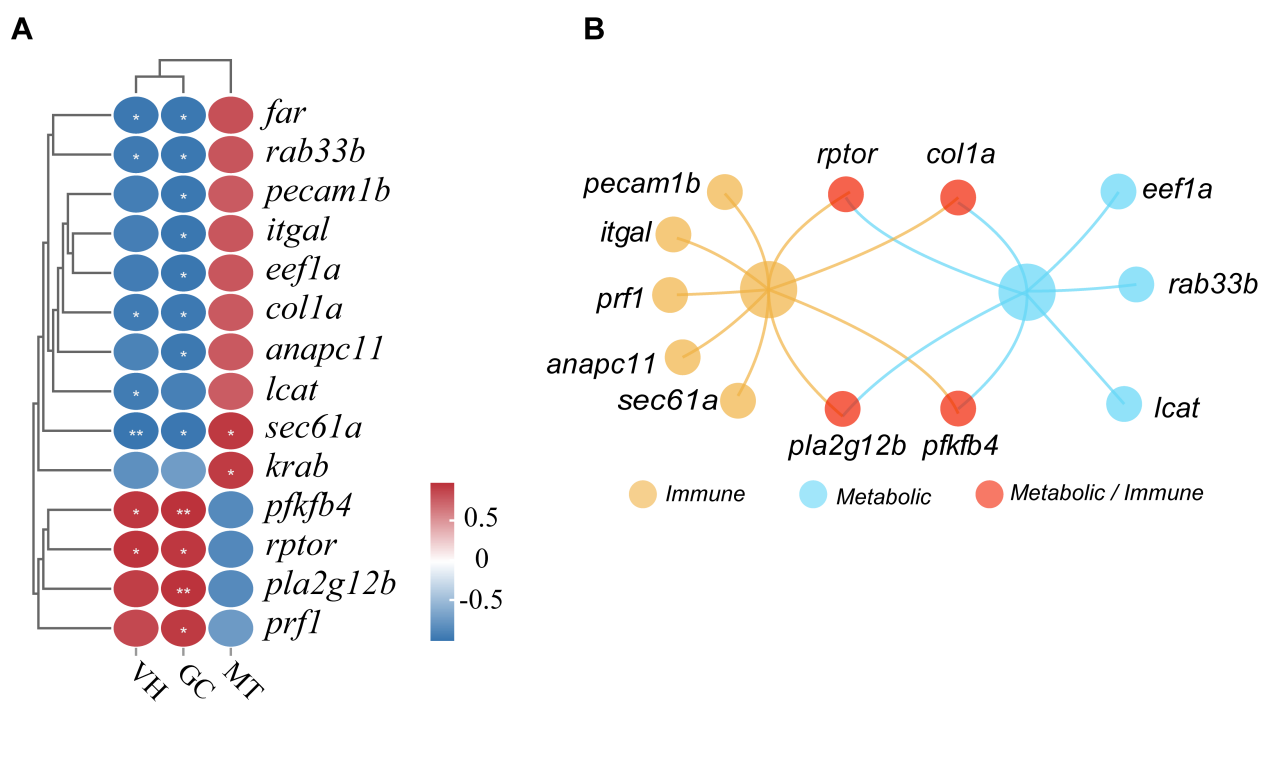


Supplementary Fig.3. (A) Association analysis between intestinal morphological traits and gene expression profiles. (B) Functional classification of genes selected from transcriptomic analysis. Light purple nodes represent immune-related genes, light blue nodes represent metabolism-related genes, and orange nodes indicate genes involved in both immune and metabolic pathways.


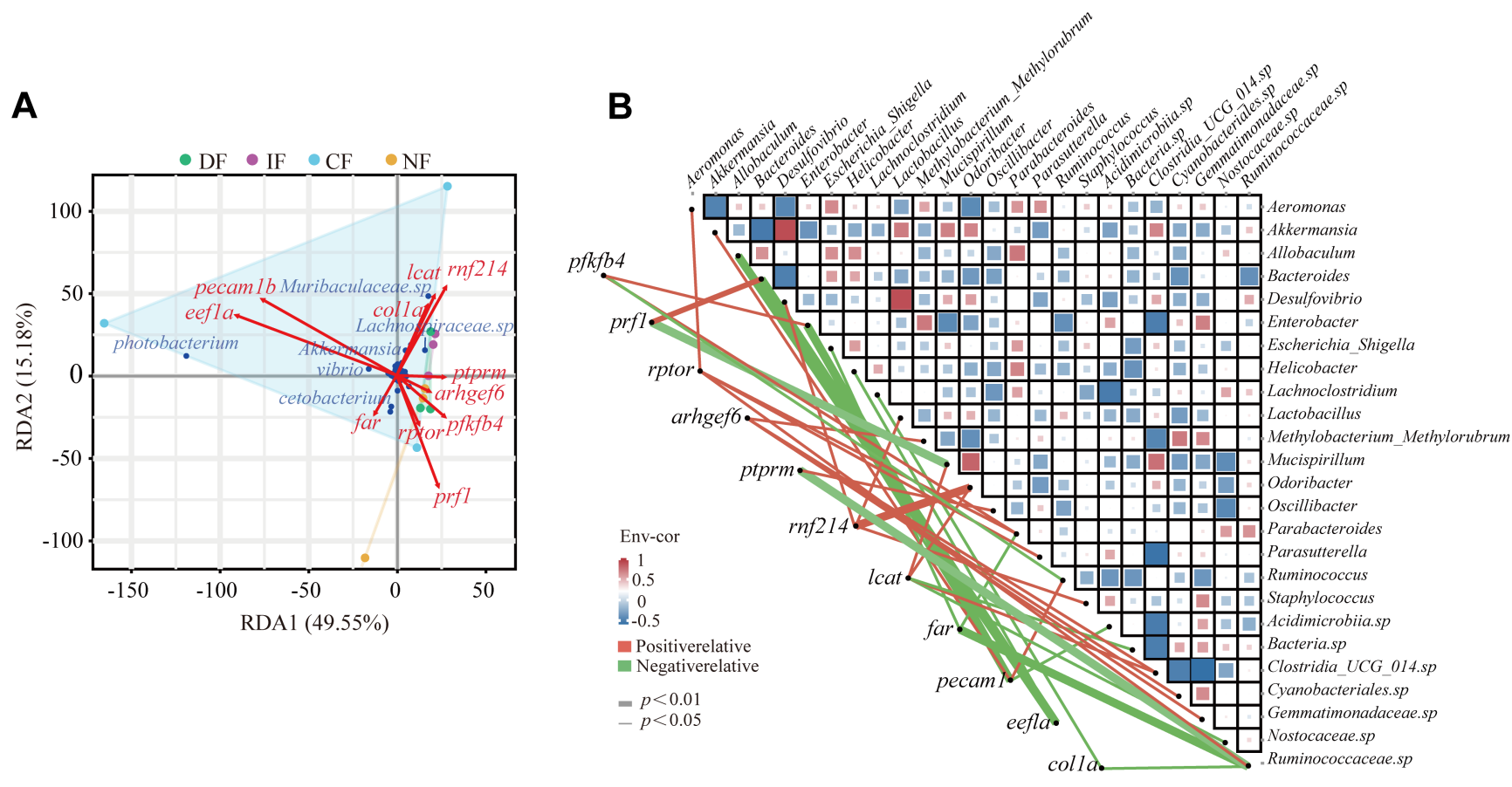


Supplementary Fig.4. RDA analysis of transcriptome and microbiota (A) and correlation heatmap (B). In the heatmap, the size of each square represents the strength of correlation between microbial taxa. Lines indicate significant correlations between microbes and genes, with red representing positive correlations and green representing negative correlations.


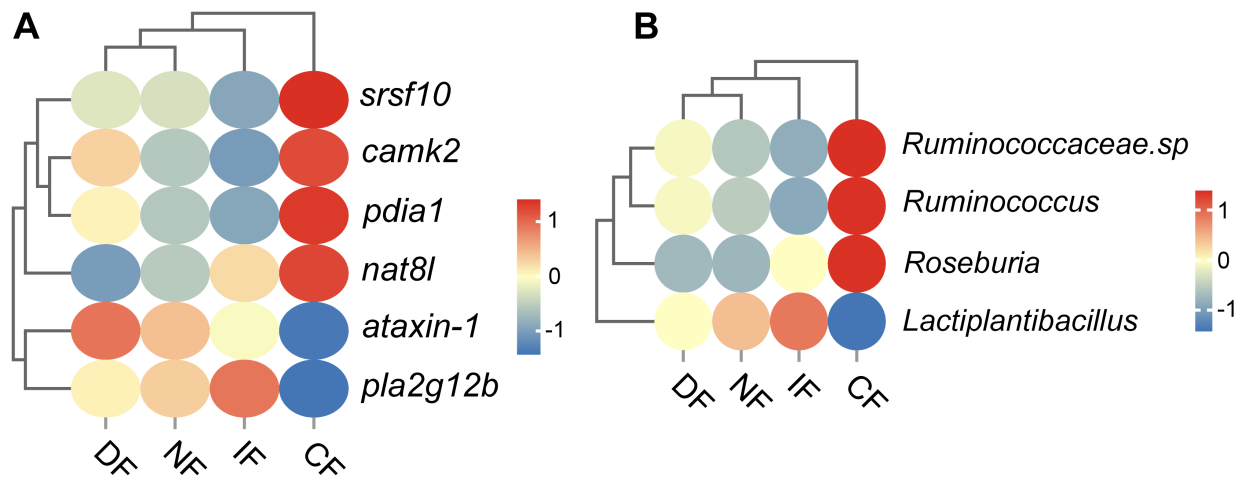
Supplementary Fig.5. Heatmap showing expression and abundance patterns of genes and microbes associated with serum biochemical indices and weight gain rate. Genes and microbial taxa significantly correlated with phenotypic traits were selected, and their relative expression (A) and relative abundance (B) are displayed across experimental groups.
